# Binding Characteristics of Sphingosine-1-Phosphate to ApoM hints to Assisted Release Mechanism via the ApoM Calyx-Opening

**DOI:** 10.1038/srep30655

**Published:** 2016-08-01

**Authors:** Hansi Zhang, Kristyna Pluhackova, Zhenyan Jiang, Rainer A. Böckmann

**Affiliations:** 1 Computational Biology, Department of Biology, Friedrich-Alexander Universität Erlangen-Nürnberg, Erlangen, 91058, Germany

## Abstract

Sphingosine-1-phosphate (S1P) is a lysophospholipid mediator carried by the HDL-associated apoM protein in blood, regulating many physiological processes by activating the G protein-coupled S1P receptor in mammals. Despite the solved crystal structure of the apoM-S1P complex, the mechanism of S1P release from apoM as a part of the S1P pathway is unknown. Here, the dynamics of the wild type apoM-S1P complex as well as of mutants were investigated by means of atomistic molecular dynamics simulations. The potential of mean force for S1P unbinding from apoM reflected a large binding strength of more than 60 kJ/mol. This high unbinding free energy for S1P underlines the observed specificity of the physiological effects of S1P as it suggests that the spontaneous release of S1P from apoM is unlikely. Instead, S1P release and thus the control of this bioactive lipid probably requires the tight interaction with other molecules, e.g. with the S1P receptor. Mutations of specific S1P anchoring residues of apoM decreased the energetic barrier by up to 20 kJ/mol. Moreover, the ligand-free apoM protein is shown to adopt a more open upper hydrophilic binding pocket and to result in complete closure of the lower hydrophobic cavity, suggesting a mechanism for adjusting the gate for ligand access.

Sphingosine-1-phosphate (S1P) is a pluripotent, zwitterionic lysophospholipid mediator. S1P binding to one of its G protein-coupled receptors (S1P_1_-S1P_5_) in the cell membrane regulates a variety of cell and tissue functions, including vascular maturation^1^,^2^, lymphocyte trafficking^3^, barrier functions^4^, calcium mobilization^5^, cell proliferation^6^,^7^,^8^, differentiation^5^, and survival^7^. S1P is synthesized locally in most organs but also circulates with a high level in blood to fully stimulate S1P receptors^3^,^9^. Plasma S1P is transported by plasma proteins, which are comprised of ~55% high density lipoprotein (HDL), ~40% albumin, and <10% low density lipoprotein (LDL) and very low density lipoprotein (VLDL)^10^,^11^,^12^.

A previous study demonstrated that apolipoprotein M (apoM) is the direct carrier of S1P in HDL and the crystal structure of recombinantly expressed apoM in complex with S1P was solved at 1.7 Å resolution^13^. ApoM belongs to the lipocalin family characterized by an eight-stranded antiparallel *β*-barrel enclosing a ligand binding pocket for small lipophilic molecules. Human apoM is a 188-residue-long protein, synthesized primarily in liver and kidney and mainly bound to HDL^14^,^15^,^16^,^17^. The N-terminal 21 amino acid residues form a hydrophobic signal peptide which is essential for anchoring the lipoprotein to the phospholipid layer^14^,^18^,^19^. ApoM has been shown to influence lipoprotein and cholesterol metabolism^20^,^21^,^22^, atherosclerosis^20^,^21^, diabetes^23^,^24^,^25^, and rheumatoid arthritis (RA)^26^,^27^ as recently shown in a number of experimental studies. Still, the molecular mechanisms of apoM action are largely unknown. ApoM can bind different ligands including diverse fatty acids and lipids, containing 14, 16, or 18 carbon atoms in their hydrophobic tail^28^. S1P is one of the apoM ligands, its binding to apoM was shown to efficiently quench the apoM intrinsic fluorescence with an IC_50_ of 0.90 *μ*M at 345 nm^28^. This indicates that the apoM-S1P complex could play a key role in the physiological S1P pathway.

In 2011, Christoffersen *et al*.^13^ reported the crystal structure of the apoM-S1P complex. The S1P headgroup was observed to bind to the hydrophilic upper moiety of apoM while the hydrophobic chain was buried in the hydrophobic lower cavity of the protein, similar to the before reported position of myristic acid in apoM^28^. The N-terminal signal peptide of apoM, which anchors apoM into HDL, was not resolved in the structure.

In apoM, S1P binds at the center of the calyx-like binding pocket through direct hydrogen bonds with Arg98, Trp100, Arg116, and Glu136, as well as via water-mediated hydrogen bonds to Tyr102, Arg143, and Tyr147^28^. The recognition of the phosphate group of S1P by the arginines appears to be highly specific. Despite the crystal structure of the apoM-S1P complex, the biological process of S1P (un-)binding to the carrier is still widely debated.

Using atomistic molecular dynamics (MD) simulations, we characterized the human-apoM binding pocket for the ligand S1P and the changes in S1P anchoring to apoM upon mutating residues involved in the polar S1P binding. Enforced S1P dissociation molecular dynamics (MD) simulations^29^ and potential of mean force (PMF) calculations for S1P unbinding were applied to shed light on the mechanism of ligand unbinding. The observed high binding free energy for S1P binding to apoM of >60 kJ/mol and the dominant interactions of polar apoM residues to binding suggest that the S1P release depends on the interaction of the apoM calyx opening with other molecules. The interactions between S1P and apoM attached to a lipid bilayer displayed similar characteristics as those in solution.

## Results

### Protonation of S1P

Possible protonation changes of S1P upon binding to apoM were addressed by analyzing the pK_a_ of S1P both in its free and apoM-bound states, as well as during unbinding from apoM.

During a 200 ns MD simulation, the pK_a_ values of the apoM-bound S1P phosphate oxygens were stable with values of ≈4, supporting the chosen protonation of the phosphate group (PO_4_^2−^). Additionally, the pK_a_ value of the amino group fluctuated around 14, consistent with the protonated state of NH_3_^+^. The total charge of bound S1P is thus −1 e, which is in line with a previous study^30^.

The pK_a_ values of an isolated solvated S1P molecule were calculated to be ≈6 for the phosphate oxygens and 11 for the amino group. Taken together, this change in S1P protonation upon solvation sums up to a net release of 0.16 protons during S1P unbinding from apoM at pH 7 (see Equation 3), i.e. the assumption of an unchanged total charge of −1 e for S1P during unbinding is well justified.

The change in pK_a_ was furthermore followed during the enforced unbinding simulation (see Supplementary Fig. S2). The change in the S1P pK_a_ values correlates with the rupture of intermolecular hydrogen bonds between apoM and S1P. The isoelectric point (pI) of apoM increased from 6.2 to 6.7 during S1P unbinding. This estimated change in pI by 0.5 pH units is in very good agreement with the experimental data reporting a decrease of the pI by 0.2 pH units upon S1P addition^28^.

### Characteristics of S1P binding to apoM

**Figure 1:**
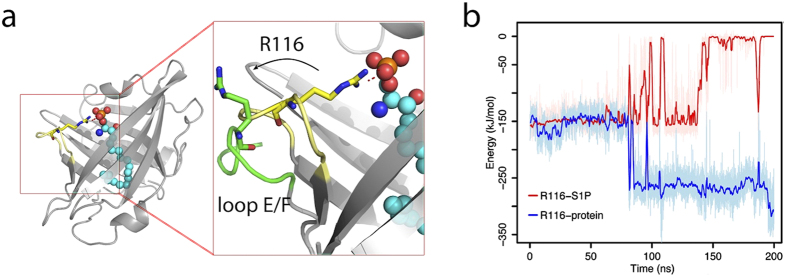
(**a**) The conformational switch of Arg116 after 80 ns of simulation time. (**b**) The interaction energy computed as the sum of short range Lennard-Jones and electrostatic interactions between Arg116 and S1P (red) and between Arg116 and the protein (blue).

The wt apoM structures both in solution and attached to POPC remained stable on the 200 ns timescale with a root mean square deviations (RMSD) of ≈2 Å (see below). ApoM overall kept its local structure with the exception of *β*-sheet E both in solution and attached to POPC. The short *β*-sheet E region (six residues) partially unfolded, thereby increasing the solvent exposure of the E/F loop. Noteworthy, the hydrogen bond between S1P and Arg116 that is located in the E/F loop was lost (Fig. 1a). However, S1P was still stable within the binding pocket. Arg116 showed a large flexibility, increasing its interaction with the rest of the protein (Fig. 1b). Due to this flexibility, Arg116 is suggested to easily adapt to different ligands and to be involved in the recognition of S1P and possibly also of interaction partners of apoM.

**Figure 2:**
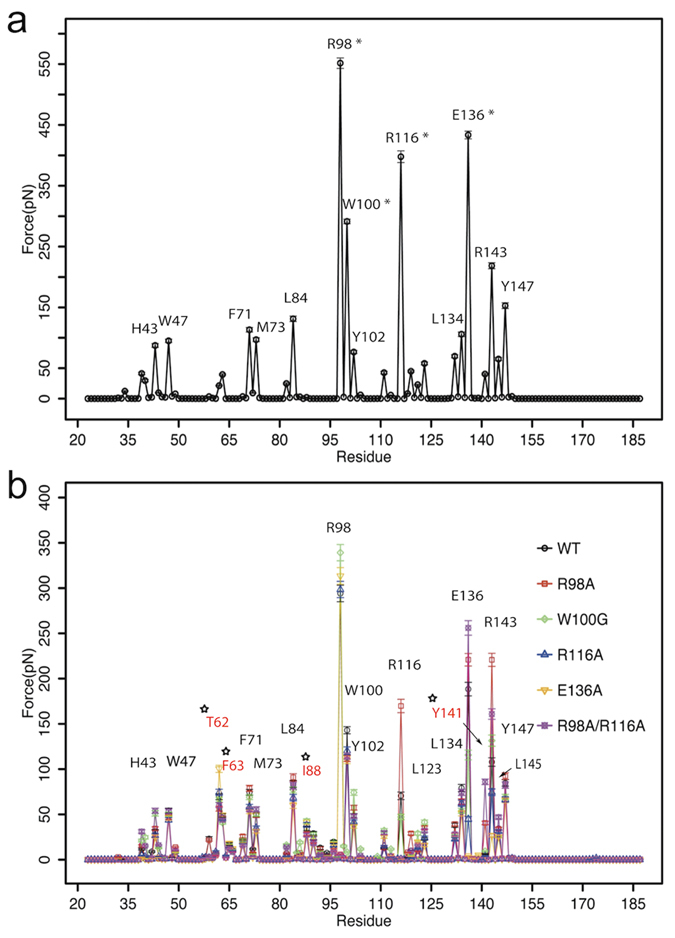
Pairwise interaction forces between apoM and S1P. (**a**) Interaction forces for the apoM-S1P complex. The pairwise forces between S1P and each residue of apoM were calculated using the force distribution analysis (FDA, see Methods). The forces were averaged over simulations of the apoM WT both in solution and anchored to POPC. The residues with largest forces (>70 pN) are labeled. The four residues with the highest values (marked by asterisk) are in accordance with the S1P binding residues reported in the crystal structure. (**b**) Interaction forces during enforced S1P unbinding. Each line was averaged over three pulling simulations for each complex. Residues with forces >40 pN are marked. Residues with forces significantly exceeding those from wt equilibrium MD simulation are labeled in red and marked with an asterisk.

**Figure 3:**
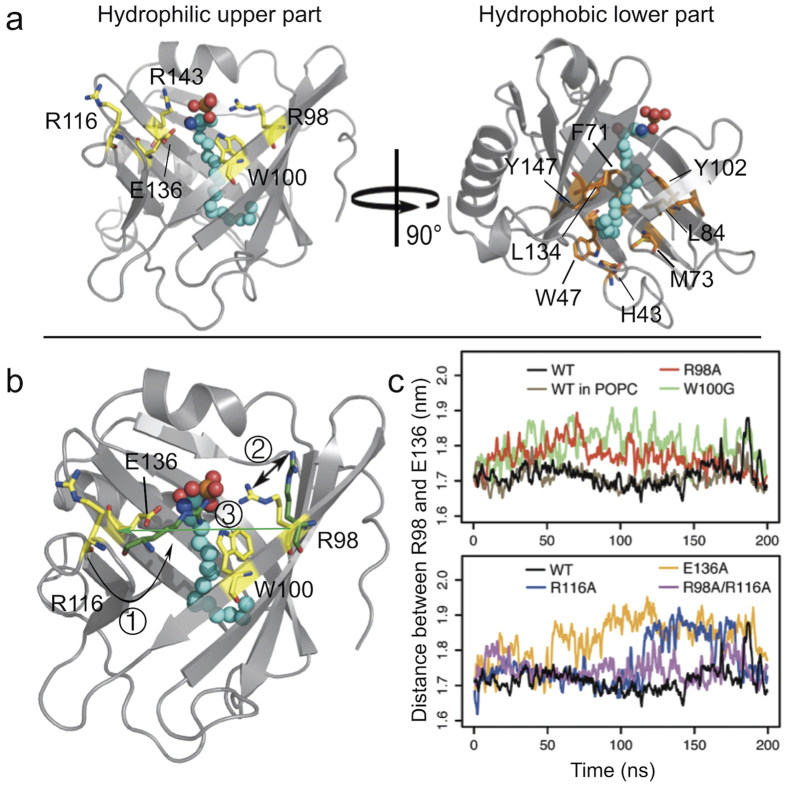
The S1P binding pocket of apoM. (**a**) A snapshot of wt apoM-S1P after 200 ns of equilibration. Left panel: The residues highlighted in yellow mainly contribute to the upper hydrophilic part of the binding pocket and stabilize the charged moiety of S1P. Right panel: The residues colored orange form the hydrophobic binding groove of apoM. (**b**) Three main conformational changes were observed for the equilibrium MD simulations of the studied mutants (colored green):① Arg116 switched to stabilize S1P in the R98A mutant, and occasionally also in the W100G and E136A mutants; ② The flexibility of Arg98 increased in the W100G mutant due to the loss of the cation-*π* interaction; ③ The distance between C_*α*_ atoms of residues 98 and 136 indicating calyx structure loosening increased for all mutants as compared to the WT protein. (**c**) The distances between C_*α*_ atoms of residues 98 and 136 as a function of simulation time for the WT (black), the WT anchored to a POPC bilayer (brown), the R98A (red), W100G (green), R116A (blue), E136A (orange), and R98A/R116A mutants (magenta).

The S1P binding environment was characterized by computing the averaged residue-wise forces between the ligand and apoM residues. Residues strongly contributing to S1P binding are easily identified by their large interaction forces (>70 pN, see Fig. 2a, visualized in Fig. 3a). The largest forces were recorded for the residues Arg98, Trp100, Arg116, and Glu136. These residues are located at the opening of apoM^13^ and interact mainly electrostatically with S1P. In the crystal structure, Arg143 was reported to form a hydrogen bond with S1P via a water molecule^13^. During the simulation, Arg143 moved closer to S1P and formed a direct hydrogen bond with the hydroxyl group of S1P. This position of Arg143 is stabilized by backbone hydrogen bonding to Glu136 (Fig. 3a, left). Residues His43, Trp47, Phe71, Met73, Leu84, Tyr102, Leu134, and Tyr147 primarily form the hydrophobic lower binding pocket for the hydrocarbon chain of the substrate (Fig. 3a, right). These interactions with S1P are accordingly dominated by Lennard-Jones interactions.

**Figure 4:**
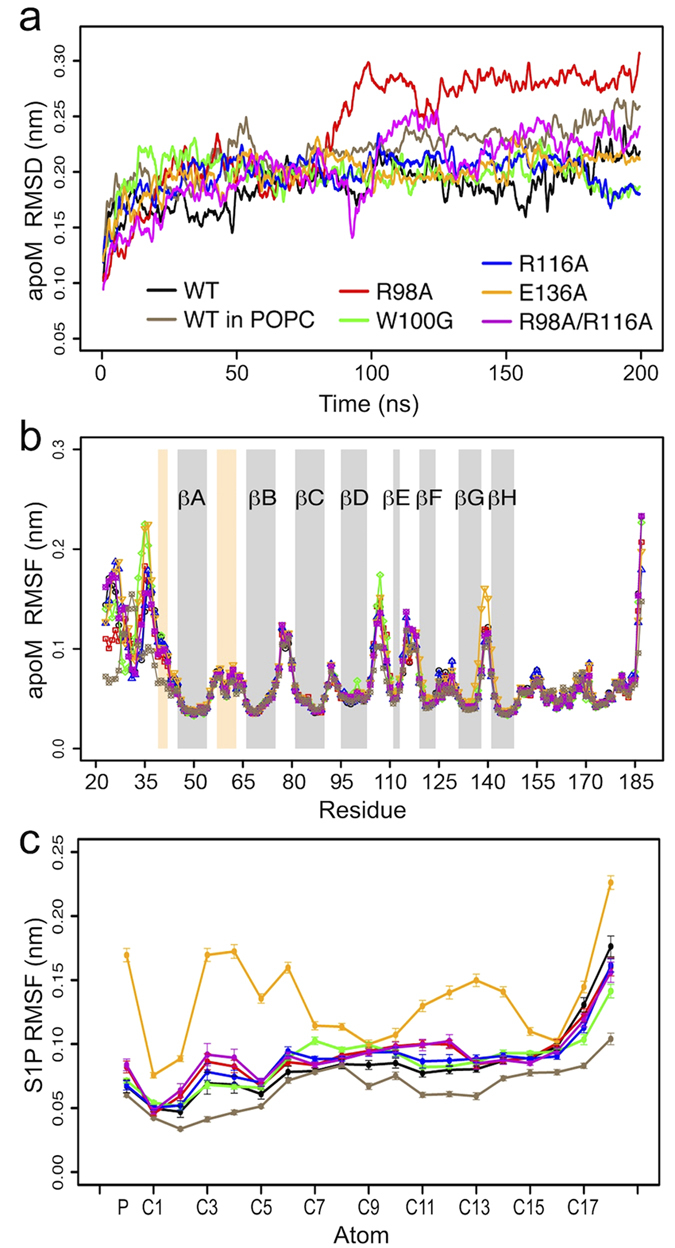
Stability and fluctuations of apoM and S1P. RMSD (**a**) and RMSF (**b**) values of apoM were analyzed for the C_*α*_-atoms of the seven studied complexes (WT, black; WT attached to POPC, brown; R98A, red; W100G, green; R116A, blue; E136A, orange; R98A/R116A, magenta). The values were averaged over 40 windows of 5 ns length each. Error bars show the standard error. (**c**) RMSF values of the phosphorus atom and carbon atoms of S1P. The values were averaged over 20 windows of 10 ns length each. Error bars show the standard error.

In all five mutant equilibrium simulations, the RMSD values were smaller than 3 Å (Fig. 4a) and the protein secondary structure was maintained (apart from partial unfolding of the *β*-sheet E, not shown) similar to the wt simulation. Large deviations as well as large fluctuations (root mean square fluctuation, RMSF) were observed for the loop regions (Fig. 4b). For the R98A mutant, Arg116 replaced Arg98 in stabilizing the phosphate group of S1P (no conformational switch as discussed above for the WT). I.e., S1P may be stabilized in the binding groove of apoM either by Arg98 or Arg116 (preferentially Arg98). In the W100G mutant, hydrogen bonding between Arg98 and S1P was slightly diminished as compared to the wt (hydrogen bond existence time decreased from 97% to 88%). This suggests that Trp100 can steady Arg98 in its binding position through a cation-*π* interaction, thus stabilizing the ligand binding. The width of the binding pocket slightly increased for all mutants as evidenced by the increased distance between the C_*α*_ of residues 98 and 136 for the mutant simulations (Fig. 3c). This loosening of the calyx structure resulted in significant reduction of the S1P binding strength (see below). This was in particular observed for R116A and R98A/R116A as well as for E136A (connection between the Glu136 and the amine group of S1P got disrupted). The flexibility of S1P was largest in E136A, suggesting a key role of Glu136 in stabilizing the ligand motion in the binding pocket (Fig. 4c). An overall reduced S1P flexibility is seen for the membrane-bound form of apoM.

### ApoM-S1P complex associated with lipid layer

**Figure 5:**
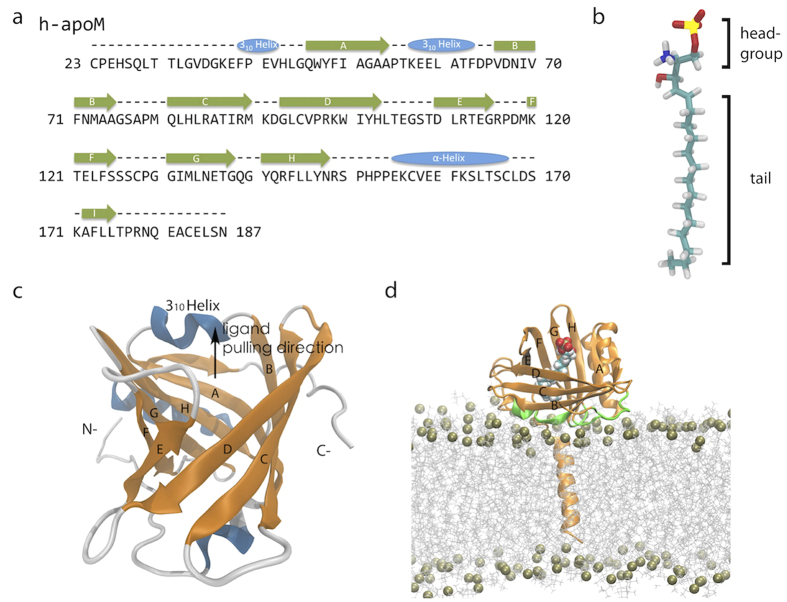
(**a**) Sequence of human apoM along with the secondary structure elements gained from the crystal structure. (**b**) S1P structure. Oxygen atoms, phosphorus atom, and nitrogen atom are shown in red, yellow and blue, respectively. (**c**) ApoM structure from residue 27 to residue 187, containing a *β*-barrel with eight anti-parallel *β*-strands displayed in cartoon representation. (**d**) The S1P-bound apoM embedded in a POPC bilayer by the N-terminal anchor helix. The region of apoM interacting with the bilayer is highlighted in green.

ApoM was suggested to be associated via the N-terminal signal peptide to HDL particles^21^. Sevvana *et al*. modeled the anchoring and suggested that hydrophobic residues facing the HDL lipid layer, including L2 (76-GSAPM), L6 (124-FSSSCPGG), L8 (150-SPHPP), and a patch (29-LTTLGV) at the N-terminus, further facilitate the interaction between apoM and HDL^28^. Here, in the apoM-membrane simulation, the loop of apoM before *β*-sheet A (41-EVHLGQW), L6 (128-CPG), L8 (150-SPHPP), and a C-terminal patch (178-RNQEAC) (Fig. 5d, green color) interacted with the POPC bilayer, similar to the suggested model. The hydrophobic residues interacted with the lipid chains, while the charged residues were engaged in interactions with the phosphatidyl choline headgroup of POPC, which is a major constituent of HDL^31^. Despite this association, the apoM-S1P complex behaved similarly to that in solution, which suggests an HDL-independent ligand binding and release mechanism. A previously suggested release of S1P due to the flexibility of the peptide segment preceding *β*-strand A appears unlikely, the flexibility of this segment was decreased for the membrane-bound apoM as compared to free apoM (Fig. 4b), and an uncovering of the bottom of the calyx was not observed in the simulations.

### Enforced S1P unbinding

The un-/binding process of S1P to apoM was addressed in enforced unbinding simulations by pulling the ligand out of apoM (compare Fig. 5). A constant velocity was applied to a spring attached to S1P (the pulling force, apoM-S1P interaction energy, and number of hydrogen bonds during unbinding are provided in Supplementary Fig. S3). In all pulling simulations, interactions to apoM were lost for distances between the phosphorus atom of S1P and the center of mass (COM) of apoM above 2.30 nm. Upon pulling, the RMSD of the C_*α*_ atoms of apoM remained below 3 Å for all systems (not shown), i.e. the protein did not undergo larger conformational changes related to S1P unbinding. Key residues involved in the unbinding process were identified using the force distribution analysis (FDA) analysis^32^: Comparison to equilibrium simulations (see Fig. 2a) shows that forces between S1P and the residues Thr62, Phe63, Ile88, and Tyr141 were markedly increased during S1P unbinding (Fig. 2b, labeled red). In turn, these residues are probably also involved in ligand recognition.

**Figure 6:**
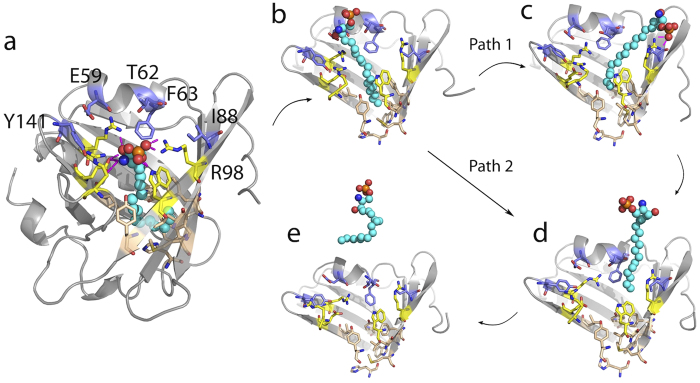
S1P unbinding process from WT apoM. The residues located in the hydrophobic lower part are colored wheat. The five residues forming the hydrophilic entrance for S1P are colored yellow. Residues interacting strongly with S1P during enforced unbinding are colored violet. Residues 23–46, 74–83 and 101–126 were omitted in figures (**b**–**e**) for clarity.

Five steps can be distinguished for the S1P unbinding pathway (compare Fig. 6): In the initial stage (stage a), the pulling force increased until reaching F_max_ (Supplementary Fig. S3), the so-called *unbinding force*. The height of this force typically depends on the pulling or unbinding velocity^33^,^34^. Concomitant with the main force barrier the number of hydrogen bonds between S1P and apoM drastically decreased (Fig. S3), suggesting the polar interactions between S1P and the hydrophilic opening of the calyx as main opposing force to unbinding.

Following the initial breakage of hydrogen bonds to the S1P headgroup, the motion of the phosphate headgroup was temporarily restricted by interactions with Thr62, Phe63, and Glu59 in the flanking 3_10_ helix (step *b*). Simultaneously, the hydrocarbon chain of S1P left the lower hydrophobic cavity. Finally, S1P binding is restricted to interactions between the hydrocarbon tail and residues on the top of apoM, especially from the flanking 3_10_ helix (*d*). In a different pathway, a prior flipping of the S1P headgroup to Arg98 was observed (*c*). In the final step, the S1P tail glided out of the apoM binding groove without exhibiting any further significant energetic barriers (*e*).

**Figure 7:**
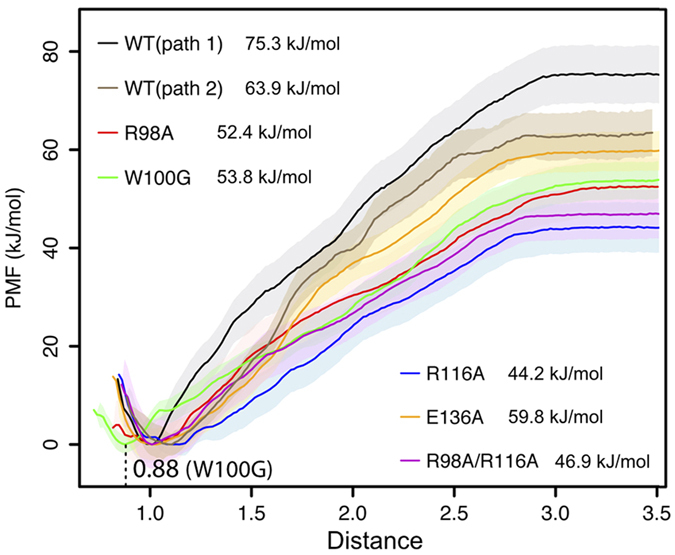
Potential of mean force (PMF) for S1P unbinding. The PMF was calculated from between 42 and 88 umbrella simulations for each system.

The potential of mean force (PMF) was analyzed for both pathways, i.e. *a*–*b*–*c*–*d*–*e* (path 1) and *a*–*b*–*d*–*e* (path 2), using umbrella sampling simulations. The PMF analysis followed the local samplings along each pathway, without restraints on the sampling orthogonal to the pulling direction. The degree of orthogonal sampling is displayed in Supplementary Fig. S4. Slightly different regions in phase space are sampled for the paths, differing in particular for the intermediate binding to Arg98 along path 1. The PMF curves for both pathways show a monotonous increase (Fig. 7), the energetic barrier for path 1 was determined to 75 kJ/mol and for path 2 to 64 kJ/mol.

The chosen apoM mutations in particular impacted the electrostatic contribution to S1P headgroup binding. For all mutants, unbinding of S1P followed the energetically favored path 2. In agreement with the selection of mutants to decrease the S1P binding affinity, the PMFs of all mutant complexes for S1P unbinding were significantly decreased by 4 kJ/mol (E136A) to 20 kJ/mol (R116A) as compared to wt apoM. In W100G, Arg98 lost the cation-*π* interaction with Trp100, resulting in weakened ligand binding (by 10 kJ/mol). Additionally, S1P was observed to interact with Tyr102 (side chain directly beneath Trp100), resulting in a shift of the lowest energy state of S1P by about 1 Å into the apoM interior. This finding suggests that Trp100 is important for keeping a certain binding position of S1P within apoM. The largest reduction of binding strength was caused by the R116A and R98A/R116A mutations. Though Arg116 is flexible it is indispensable to integrate the binding pocket in the calyx structure.

### ApoM microenvironment changes during ligand unbinding

Sevvana *et al*. reported the distance of the broadest opening of the fatty-acid-bound-apoM to 29 Å (distance between the C_*α*_ atoms of Gly93 and Tyr141), tapering to 19 Å in the middle of apoM (Lys99 to Phe144) and 20 Å at the bottom (His103 to Tyr147)^28^. The corresponding values in the equilibrated S1P-bound apoM wild type complex are in close agreement with the reported data. However, following S1P removal both the diameter of the opening of the calyx increased by ≈2 Å as well as the flexibility in particular of the middle region of the barrel (see Supplementary Fig. S5). The structure of the ligand-free mutants resembled the one of the ligand-free apoM structure.

The two tyrosine residues in positions 102 and 147 separate the hydrophobic lower cavity from the hydrophilic upper portion of the binding pocket. The minimum distance between Tyr102 and Tyr147 exhibited a slight decrease during the S1P pulling simulation, hindering the ligand from release (data not shown). After the tail of S1P left the hydrophobic pocket, these two residues restored their positions and showed an increased flexibility as compared to the S1P-bound state (Supplementary Fig. S4). This indicates that the two tyrosines form a gate, similar to the arrangement of the corresponding residues Tyr83 and Tyr131 reported for the human complement protein C8*γ*^35^.

### ApoM cavity volume

**Figure 8:**
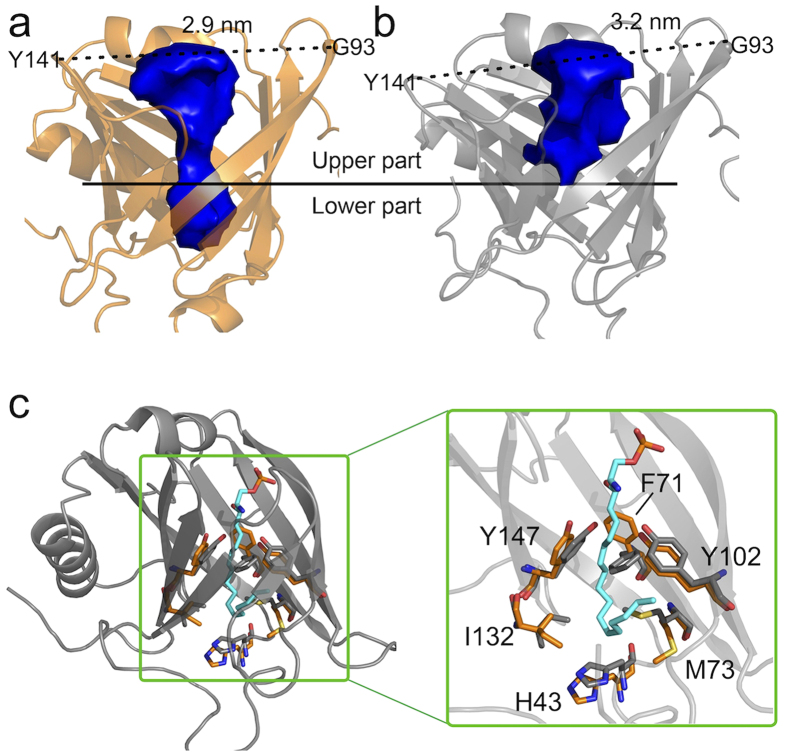
S1P binding pocket of apoM. (**a,b**) The binding pockets of S1P-bound apoM (orange) and of ligand-free apoM (grey) are compared to each other. The blue surface borders the measured pocket volumes. The upper pocket, as well as the distance between the C_*α*_ atoms of residues G93 and Y141 are increased for the ligand-free apoM while the lower part of the cavity is closed. (**c**) The gating is achieved by Tyr102 and Tyr147 and the pocket occluded by Phe71, Met73, His43, and Ile132 (grey sticks). For bound S1P (cyan sticks), the sidechains (orange sticks) slightly reorient to accomodate the ligand.

The opening of the calyx as well as the above reported gating of the tyrosines 102 and 147 influenced the form and size of the apoM binding pocket. The total volume of the binding pocket is ≈430 Å^3^ in the S1P-bound crystal structure, which may be divided into an upper part with ≈285 Å^3^ and a lower pocket with ≈145 Å^3^ (gate formed by residues Tyr102 and Tyr147, see Fig. 8 & Supplementary Table S1). Both simulations of ligand-free apoM, i.e. a simulation starting from the crystal apoM after S1P deletion and a simulation of apoM after enforced S1P unbinding, showed a widened opening and a significantly increased pocket volume of ≈455 Å^3^ for the upper cavity, while the lower part was completely closed due to rearrangement of the hydrophobic residues Phe71, Met73, Ile132, and His43 (Fig. 8c): The re-oriented Phe71 (Fig. 3a) contributed most to the shielding of the binding cavity from the solvent, a function that has been observed in other lipocalins as well (e.g. in bovine odorant-binding protein^36^ and in aphrodoisin^37^). Our results suggest that the empty apoM structure adopts a more open conformation that is prone to ligand recognition and binding. Preceding ligand binding, the hydrophobic lower cavity is shielded.

The bottom part of the calyx is formed by His43 and Trp47. His43 is replaced by phenylalanine in some other lipocalins with the side chain pointing either towards the inside of the calyx or positioned parallel to the bottom of the calyx. Here, His43 adopts the latter conformation in the apoM-S1P complex, enabling to accomodate the long S1P hydrocarbon chain within the calyx (Fig. 8c). In both ligand-free and ligand-bound complexes, His43 was very stable and close to Trp47, which forms *π*-*π* interaction with His43 and cation-*π* interaction with Arg149 to stabilize the bottom of the calyx. It was suggested before that Trp47 stabilizes the lipocalin fold and supports ligand binding^28^,^38^.

## Discussion

S1P is a lipidic mediator in mammalian bodies. In order to be able to bind to its receptor and trigger the signaling pathway it has to be first transported by its carrier apoM protein to the GPCR. For receptor binding and activation, S1P unbinding from the carrier apoM is required first. Here, we characterized by means of MD simulations the S1P binding strength to wt apoM as well as to a number of apoM mutants and investigated the plasticity of the apoM carrier upon S1P dissociation.

The apoM binding pocket was seen to be gated by the tyrosines in positions 102 and 147. These tyrosines were observed to be more flexible in the ligand-free apoM, shielding together with Phe71 the lower hydrophobic cavity of apoM in the apo state. A similar rearrangement for closure was seen for the hydrophobic amino acids lining this cavity.

The upper part of the binding cavity, which opens significantly in the absence of the ligand, is mostly formed by hydrophilic residues. These play an important role in stabilizing the charged S1P headgroup in the bound state. The unbinding energy for S1P was estimated to more than 60 kJ/mol. It was determined along a path through the calyx opening at the top of apoM. Mutation(s) of apoM residues anchoring the S1P headgroup (Arg98, Trp100, Arg116, and Glu136) decreased the potential of mean force by up to 20 kJ/mol. Thus, the main energetic barrier for S1P unbinding was due to hydrogen bonding of the polar S1P headgroup. Our results moreover suggest that the 3_10_ helix is involved in ligand recognition upon binding.

ApoM was found to be stably anchored in the POPC bilayer via the N-terminal signal peptide and by interactions between the loops at the bottom of the calyx and the lipid bilayer. Interestingly, the anchoring of apoM to a lipid bilayer did hardly affect the S1P bound conformation or ligand unbinding from apoM, suggesting an HDL-independent ligand binding mechanism. Previously discussed alternative ligand release mechanisms either through the bottom of the calyx or a lateral release between strands D and G^13^ are unlikely given the overall structural stability of apoM observed in the simulations.

In summary, our results suggest (i) that S1P binds to and leaves apoM via the calyx opening, and, (ii) that a specific mechanism decreasing the energetic barrier is essential for the transfer of S1P to the S1P receptor. This could be achieved through other proteins, like the endothelial lipase that was suggested to mediate S1P release from HDL^39^ or by direct binding of apoM to the S1P_1_ receptor.

## Methods

### Starting Structures

All molecular dynamics simulations of apoM in complex with S1P were based on the crystal structure (PDB ID 2YG2^13^). Not resolved residues were built using the PyMOL^40^ software (see Fig. 5a for the apoM sequence). The total complex comprised 165 residues of apoM (Fig. 5c) and one S1P molecule (Fig. 5b).

Based on the crystal structure, four residues (Arg98, Trp100, Arg116 and Glu136) of apoM can form direct hydrogen bonds to S1P via their side chains. Here, five mutants (R98A, W100G, R116A, E136A, and the double mutant R98A/R116A) were designed in order to study the influence of the specific S1P headgroup binding on the total binding free energy.

Additionally, the N-terminal membrane anchor region of apoM, ‘1-MFHQI WAALL YFYGI ILNSI YQ-22’, was modeled in *α*-helical conformation using PyMOL and inserted into a lipid bilayer by the tool ‘g_membed’^41^ (Fig. 5d). The membrane consisted of 332 1-palmitoyl-2-oleoyl-sn-glycero-3-phosphocholine (POPC) molecules mimicking the HDL lipid layer.

### Force field parameters

All simulations were performed with the GROMACS 4.5 package^42^ using a combination of the AMBER99sb-ildn protein force field^43^ and the Slipids force field for S1P and POPC^44^,^45^,^46^. Force field parameters for the S1P molecule were prepared based on the parameters for sphingomyelin available within the Slipids force field by deleting the tail connected to the nitrogen atom. The nitrogen atom was accordingly changed into a 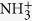 group. In order to keep the charges for this amino group equal to the amino group of phosphatidylethanolamine (PE) in the Slipids force field, the charge of the adjacent carbon (C2) had to be altered from 0.09 e to 0.07 e.

In the crystal structure, the phosphate group of S1P forms an intramolecular hydrogen bond with Arg98 of apoM. Shin *et al*. reported that the phosphomonoester of PA is deprotonated upon formation of a hydrogen bond with the primary amine of the headgroup of PE lipids, or to arginines and lysines of proteins^30^. These findings suggest that the phosphate group of S1P should be modeled with a charge of −2 e (PO_4_^2−^) if bound to apoM. This assumption is further validated by pK_a_ calculations for S1P (Results Section). Overall, S1P thus has a total charge of −1 e. Charges of the phosphate group were gained from restrained electrostatic potential (RESP) calculations^47^ using Gaussian09^48^. In detail, the charges of methyl phosphate (CH_3_-PO_4_^2−^) were calculated using the Hartree-Fock method with the 6-31G* basis set as is typical for charge estimation in the Amber force field^49^. The charges of chemically equivalent oxygen atoms were symmetrized. The force field parameters for S1P are summarized in Table S2 of the Supplementary Information.

### MD simulation parameters

The wild type (wt) apoM-S1P complex and selected mutants were solvated using the TIP3P water model^50^. Na^+^ and Cl^−^ ions were randomly placed in the solvent phase at a physiological concentration of 0.15 M. Additional ions were added to neutralize the system.

A 200 steps energy minimization using the steepest descent algorithm was performed, followed by a 200 ps position restrained simulation with harmonic restraints (*k* = 1000 kJ/mol/nm^2^) on all protein heavy atoms to relax the water and ions. Subsequently, the system was simulated for 200 ns at constant pressure and temperature (NpT ensemble). The temperature was controlled to stay at 310 K using the v-rescale thermostat^51^ with a coupling time of 0.1 ps. The pressure was kept constant at 1.013 bar by applying an isotropic coupling using the Parrinello-Rahman barostat^52^,^53^ with a coupling time of 4 ps. All bonds were constrained using the LINCS algorithm^54^,^55^. Short-range interactions were cut-off at 1 nm, and the long-range electrostatic interactions were calculated using the Particle Mesh Ewald (PME) method^56^,^57^. An integration timestep of 2 fs was used in all simulations. The equilibrated apoM-S1P structure was used to prepare the mutant structures. The same simulation procedure was used for the equilibrium MD simulations of the five investigated mutants.

For the simulation of apoM anchored to a POPC bilayer, the same simulation parameters were used except for the coupling set. Here, the temperature was coupled using the Nosé-Hoover thermostat^58^,^59^ with a coupling time of 0.5 ps and the pressure was kept constant at 1.013 bar by applying a semi-isotropic coupling using the Parrinello-Rahman barostat^52^,^53^ with a coupling time of 10 ps.

### Enforced S1P Unbinding and Umbrella Sampling

Force probe simulations or steered molecular dynamics (SMD) simulations^33^,^60^ are frequently employed to study unbinding processes or steered conformational changes in proteins.

Here, an external force was applied to the phosphorus atom of the S1P headgroup to drive the S1P unbinding from apoM. In order to avoid co-pulling of apoM, the protein was kept in place by keeping the positions of the apoM residues Gln81 and Gly131 fixed. These are located at the bottom of apoM with side chains pointing outwards and are thus expected not to influence S1P binding. No restraint was applied for apoM anchored to a POPC bilayer.

The spring attached to S1P was pulled at a constant velocity of 0.01 nm/ns (spring constant *k* = 100 kJ/mol/nm^2^), applied along the preset pulling direction, i.e. the axis of the binding pocket (see Fig. 5c). Motion of the ligand orthogonal to the pulling direction was not restricted.

During enforced unbinding, the total distance between the phosphorus atom of S1P and the center of mass (COM) of apoM increased from 1 nm (bound state) to 3.3 nm (free state). In order to grasp possibly varying unbinding pathways, the enforced unbinding of each mutant was repeated three times.

The potential of mean force (PMF) for ligand unbinding was calculated using umbrella sampling along the S1P unbinding pathway. As start structures for the umbrella sampling simulations, starting structures were selected from the enforced unbinding simulations with a spacing of the phosphorus atom positions of S1P along the pulling axis of 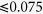 nm.

In the umbrella simulations a spring constant *k* = 1000 kJ/mol/nm^2^ was used. Each umbrella was simulated for at least 10 ns. Some samples were equilibrated for longer times in order to obtain sufficient sampling (umbrella histograms shown in Supplementary Information Fig. S1). In total, between 41 and 88 umbrella simulations were performed for each system (total of 482 umbrella simulations). The weighted histogram analysis method (WHAM, GROMACS tool *g_wham*) was used to extract the PMF from the obtained distributions^61^. The statistical error was estimated using bootstrap analysis with 200 bootstraps as implemented in *g_wham*, treating complete histograms as independent data points.

In order to study the conformational flexibility of the ligand-free apoM structure, both the wild type apoM after S1P pulling, as well as the crystal apoM structure with removed S1P, were equilibrated for 200 ns.

### pK_a_ calculation

Changes in the protonation state of S1P may be estimated from an *in silico* pK_a_ analysis. Here, the PROPKA (version 3.1) workflow^62^,^63^ was used: PROPKA determines the pK_a_ of ionizable groups through application of an environmental perturbation:


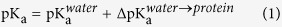



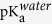
 is the pK_a_ value of the isolated titratable group in water (also often referred to as 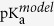), and 
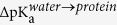
 is the protein environmental contribution to the energy difference between protonated and deprotonated forms of the amino acid or a molecule, i.e. the influence of the protein surrounding. This protein contribution predicted by PROPKA 3.1 consists of (1) desolvation effects, (2) hydrogen bond interactions (*HB*), (3) unfavorable electrostatic reorganization energies (*RE*), and (4) Coulombic interactions (*QQ*):


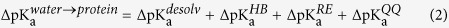


In order to estimate whether S1P dissociation from apoM results in a protonation change of S1P, the overall net change *n* of S1P was calculated based on the pK_a_ change:


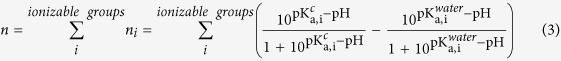


The pK_a_ values of all titratable groups (two oxygen atoms of the phosphate group and one nitrogen atom of the amino group) in the ligand were summed. 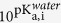 and 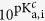 refer to the pK_a_ values of S1P in water and within the apoM-S1P complex, respectively. The isoelectric point (pI) of the protein can as well be obtained using PROPKA 3.1. The change of pI was recorded as a function of simulation time.

### Force distribution analysis

The propagation of internal strain through apoM by S1P un-/binding was followed using force distribution analysis (FDA)^32^ implemented in GROMACS 4.5.3.

The time-averaged residue-pair wise force between S1P and individual apoM residues was analyzed in order to pinpoint key residues involved in the unbinding process. The residue-pair wise force 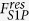 at every time point was obtained by summing up all atom-pair wise forces F_*ij*_ from all pairs of atoms *i* of S1P and *j* of each residue of apoM.

## Additional Information

**How to cite this article**: Zhang, H. *et al*. Binding Characteristics of Sphingosine-1-Phosphate to ApoM hints to Assisted Release Mechanism via the ApoM Calyx-Opening. *Sci. Rep.* **6**, 30655; doi: 10.1038/srep30655 (2016).

## Supplementary Material

Supplementary Information
