# Supplementary material for: Binding Characteristics of Sphingosine-1-Phosphate to ApoM hints to Assisted Release Mechanism via the ApoM Calyx-Opening
Source: Sci Rep. 2016 Aug 1;6:30655. doi: 10.1038/srep30655 (PMC4967915; doi:10.1038/srep30655)
Supplement: Supplementary Information [file srep30655-s1.pdf]

Supplementary Information: Binding Characteristics of Sphingosine-1-Phosphate to ApoM hints to Assisted Release Mechanism via the ApoM Calyx-Opening

Hansi Zhang[1]**, Kristyna Pluhackova**[1]**, Zhenyan Jiang**[1]**, and Rainer A. Böckmann**[1,*]

[1]Computational Biology, Department of Biology, Friedrich-Alexander Universität Erlangen-Nürnberg, Erlangen, 91058, Germany
[*]rainer.boeckmann@fau.de

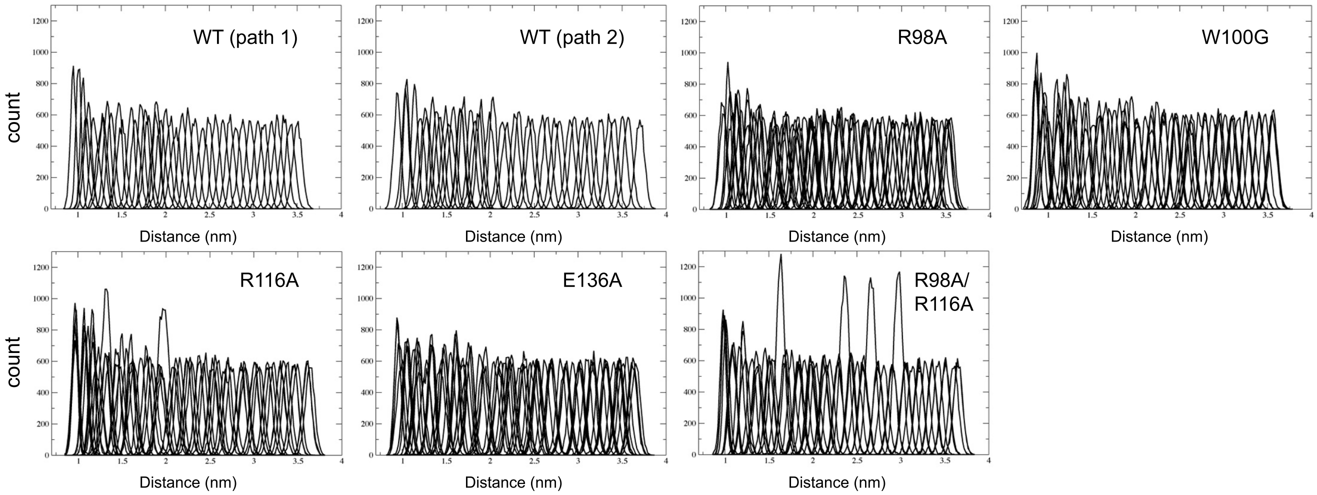

Figure S1. The histograms of umbrella sampling for the wild type and for the mutants.

Table S1. The volumes of the apoM binding pocket. The cavity volumes were computed with the program "Pocket Volume MEasurer" (POVME)[1].

System	Upper part (Å^3)	Lower part (Å^3)
S1P-bound apoM	285	145
apoM after S1P deletion	456	0
apoM after S1P pulled out	452	0

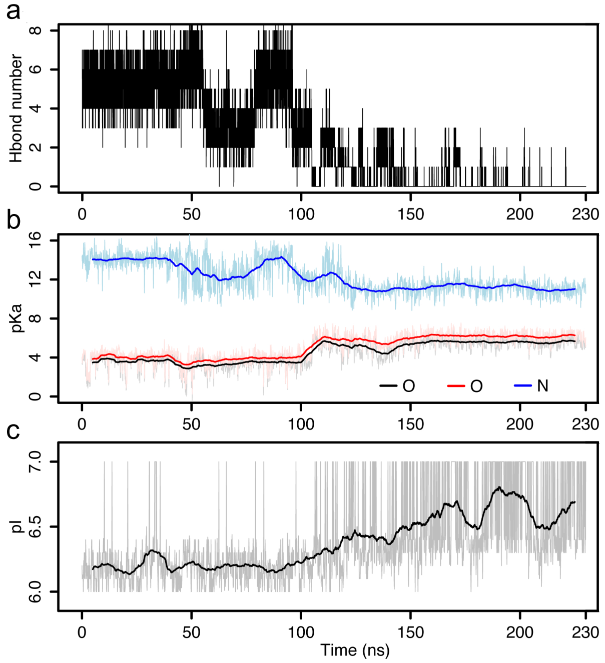

Figure S2. The evolution of the numbers of hydrogen bonds between S1P and apoM along the unbinding simulation (a). pK_a values of S1P (b), oxygen atoms from the phosphate moiety are shown in black and red, and nitrogen atom from the amino group in blue. The change of the isoelectric point (pI) of apoM during the pulling process is shown in (c).

Atom name	Atom type	Charge	Atom name	Atom type	Charge
O12	O2L	-0.951	C27	CTL2	0
P	PL	1.38	H7S	HAL2	0
O13	O2L	-0.951	H7R	HAL2	0
O14	O2L	-0.951	C28	CTL2	0
O11	OSLP	-0.622	H8S	HAL2	0
C1	CTL2	0.34	H8R	HAL2	0
HA	HAL2	-0.12	C29	CTL2	0
HB	HAL2	-0.12	H9S	HAL2	0
C2	CTL2	0.405	H9R	HAL2	0
HS	HAL2	0.09	C210	CTL2	0
N1	NH3L	-0.29	H10S	HAL2	0
H1	HCL	0.31	H10R	HAL2	0
H2	HCL	0.31	C211	CTL2	0
H3	HCL	0.31	H11S	HAL2	0
C21	CTL2	0.03	H11R	HAL2	0
H21	HAL2	0.13	C212	CTL2	0
OH1	OHL	-0.74	H12S	HAL2	0
HO1	HOL	0.47	H12R	HAL2	0
C22	CET1	-0.28	C213	CTL2	0
H22	HEL1	0.12	H13S	HAL2	0
C23	CET1	-0.09	H13R	HAL2	0
H23	HEL1	0.1	C214	CTL2	0
C24	CTL2	0.04	H14S	HAL2	0
H4S	HAL2	0.04	H14R	HAL2	0
H4R	HAL2	0.04	C215	CTL2	0.047
C25	CTL2	0	H15S	HAL2	-0.007
H5S	HAL2	0	H15R	HAL2	-0.007
H5R	HAL2	0	C216	CTL3	-0.081
C26	CTL2	0	H16S	HAL3	0.016
H6S	HAL2	0	H16R	HAL3	0.016
H6R	HAL2	0	H16T	HAL3	0.016

Table S2. Simulation parameters for S1P molecule based on Slipids parameters.

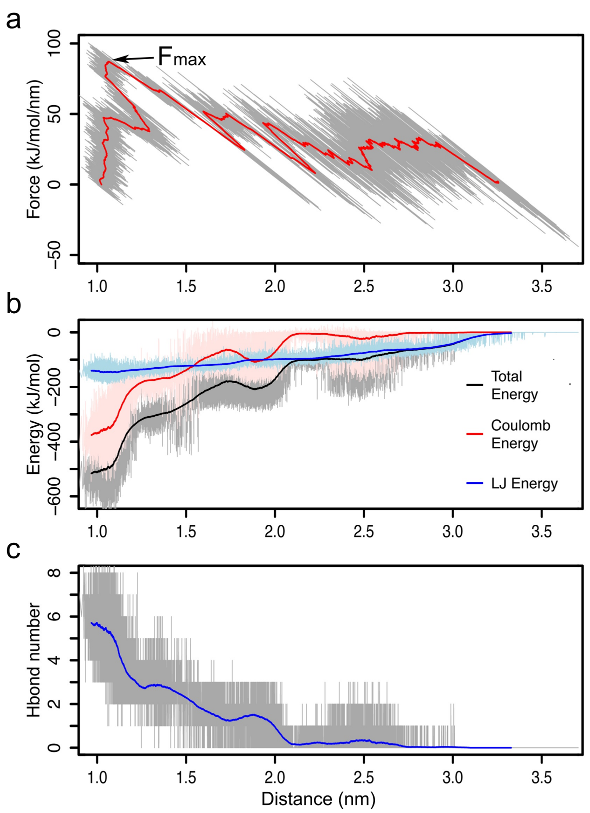

Figure S3. Enforced unbinding of S1P from WT apoM. Profiles of the applied pulling force (a), the S1P-apoM interaction energy (b), consisting of short range Lennard-Jones and Coulomb interactions, and the number of intermolecular hydrogen bonds (c) are given as a function of the distance between the S1P phosphorus atom and the COM of apoM. The smooth solid lines are moving averages of the original data.

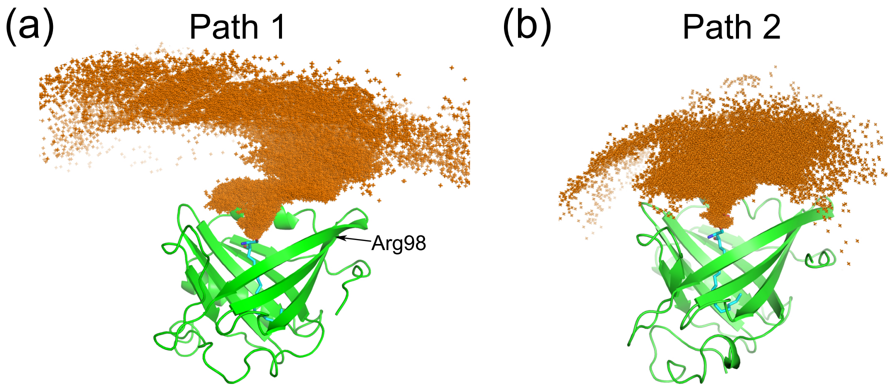

Figure S4. Unbinding positions of S1P. Overlay of the phosphorus atom positions (orange dots) of S1P from the umbrella samplings along path 1 (A) and path 2 (B), respectively. The apoM structure is shown in cartoon representation.

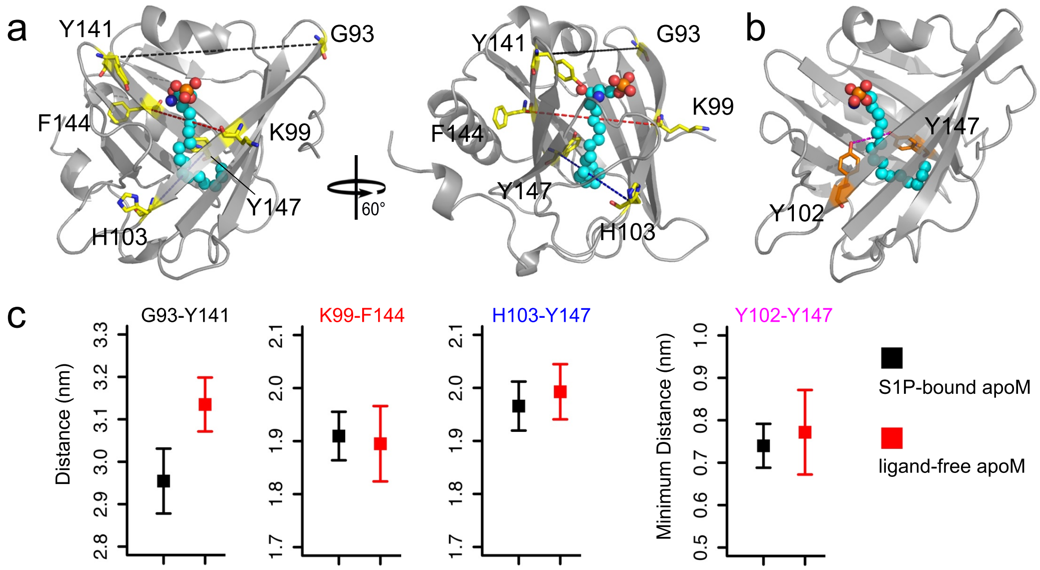

Figure S5. **Structure comparison of ligand-free and S1P-bound apoM.** (a) The three colored lines in black, red, and blue represent the distances of the opening, middle, and bottom parts of apoM, respectively. (b) The pink line shows the minimum distance between residues Tyr102 and Tyr147. (c) The average values and deviations of the distances between Cα atoms of G93-Y141, K99-F144, H103-Y147, and the minimum distance between Y102 and Y147 from the S1P-bound apoM MD simulation (black) and the ligand-free apoM equilibration simulation (red) were analyzed.

References

1. Durrant, J. D., de Oliveira, C. A. F. & McCammon, J. A. POVME: An algorithm for measuring binding-pocket volumes. *J. Mol. Graph. Model* **29**, 773–776 (2011).
